# Vectorcardiography in CRT: What We Know and What There Is to Learn

**DOI:** 10.3390/jcdd12050177

**Published:** 2025-05-07

**Authors:** Muhammet Dural, Frederieke Eerenberg, Karin C. Smits, Uyên Châu Nguyên, Kevin Vernooy, Antonius M. W. van Stipdonk

**Affiliations:** 1 Department of Cardiology, Cardiovascular Research Institute Maastricht (CARIM), Maastricht University Medical Center+ (MUMC+), 6229 ER Maastricht, The Netherlands; f.eerenberg@student.maastrichtuniversity.nl (F.E.); karin.smits@maastrichtuniversity.nl (K.C.S.); u.nguyen@maastrichtuniversity.nl (U.C.N.); kevin.vernooy@mumc.nl (K.V.); twan.van.stipdonk@mumc.nl (A.M.W.v.S.); 2 Department of Cardiology, Eskişehir Osmangazi University Faculty of Medicine, Eskişehir 26040, Turkey; 3 Department of Physiology, Cardiovascular Research Institute Maastricht (CARIM), Maastricht University, 6229 ER Maastricht, The Netherlands

**Keywords:** vectorcardiography, cardiac resynchronization therapy, QRS area, T-wave area, heart failure

## Abstract

Vectorcardiography (VCG) is an electrophysiological investigation technique, giving supplementary information about the electrical activation of the heart, compared to traditional 12-lead electrocardiography (ECG). Whereas the 12-lead ECG has found its way into global clinical cardiology practice in numerous cardiac pathophysiological instances, VCG has not. In an investigation of the electrical activation of the heart in cardiac resynchronization therapy (CRT), in order to understand the baseline pathology in potentially eligible patients, and to understand and optimize CRT-derived paced activation of the heart in the therapy’s recipients, all of these aspects are essential to the success of the therapy. Due to a consistently present group of non-responders in CRT, VCG has gained interest as a potential improvement in this field. This review comprehensively summarizes the contemporary evidence for the additional value of VCG in CRT, as well as current deficiencies in evidence, to support its implementation in global practice in addition to, or as a substitution for, traditional 12-lead ECG.

## 1. Introduction

Cardiac resynchronization therapy (CRT) improves clinical outcomes by enabling ventricular reverse remodeling in patients with dyssynchronous heart failure (HF) and reduced left-ventricular ejection fraction (LVEF) [1,2]. Since the initial implementation of CRT, QRS duration has been identified as a key marker for electrical dyssynchrony and has been selected as a main inclusion criterion in all landmark CRT trials [1,2,3,4,5,6,7,8,9,10]. Subsequent evaluation has found that patients with a longer QRS duration benefited more from CRT [11]. Soon after the initial experience of and trials in CRT, the importance of QRS morphology was recognized. Substudies of landmark trials have shown that patients with left bundle branch block (LBBB) morphology benefit the most from CRT, while patients with intraventricular conduction delay (IVCD) and right bundle branch block (RBBB) benefit little [2,3,6,12,13,14].

Ever since this, guidelines have recommended that the selection of patients for CRT be based on QRS duration and morphology [15]. However, a significant portion of patients implanted with a CRT device are deemed to derive no or insufficient benefit from it, and experts have simultaneously recognized the underuse of CRT in patients eligible for resynchronization [16]. A large part of this misuse of CRT can be explained by shortcomings in these selection criteria.

In recent years, parameters obtained from electrocardiography (ECG)-derived vectorcardiography (VCG) have attracted attention as potential markers of electrical dyssynchrony. A VCG represents the electrical activity of the heart as a vector loop in three-dimensional (3D) space, by measuring the instantaneous magnitude and direction of the combined activation (vector) from each of 12 leads from the ECG for each time-point in the cardiac cycle. The summarized 3D information in this loop logically entails information on both the duration, direction and magnitude of electrical activation (depolarization) and the relaxation (repolarization) of both the atria and ventricles. Compared to the complex interpretation of the 12-lead ECG, all information is now combined in one loop.

Recently, multiple observational studies have shown that VCG-derived parameters such as QRS area and T-wave area have great promise in delineating the true nature of ventricular conduction delay and, hence, its amenability to CRT [17,18,19]. In this review, the physiological background, existing evidence, and the gaps in evidence for the use of VCG parameters, instead of those used for a standard 12-lead ECG in CRT, are comprehensively summarized.

## 2. Limitations of Standard 12-Lead ECG

For optimal and efficient left ventricular (LV) systolic function, synchronized ventricular activation is a crucial prerequisite. In conduction abnormalities such as LBBB or right ventricular pacing, synchronous ventricular activation via the specialized conduction tissue of the heart is impossible or bypassed. In damaged tissue, conduction velocity is significantly slower than in the healthy His–Purkinje system, and the wavefront of activation changes due to the formation of fibrotic areas [20,21]. This focal or multidimensional slowing of conduction causes the prolongation of total ventricular activation visible on traditional 12-lead ECG as QRS complex prolongation. Although QRS duration, a basic indicator of delayed electrical activation, is the most used parameter in predicting the response to CRT, it does not provide information about the underlying electrical mechanism. Since QRS duration reflects total ventricular activation time, it is not a reliable marker for LV-specific activation delay, as right ventricular activation delay may cause just as much QRS duration prolongation. Additionally, structural myocardial properties, e.g., the loss or gain of myocardial mass (myocardial infarction and replacement scar versus left ventricular hypertrophy), may increase QRS duration because of focal conduction delay. Moreover, there may be up to a 20 ms variability in QRS duration measurement [22]. Therefore, interest shifted from QRS duration to QRS morphology. QRS morphology reflects the QRS pattern of the 12-lead ECG, suggestive of the underlying mechanism or location of the conduction delay. Patterns suggestive of left and right bundle branch block (LBBB and RBBB), as well as a specific intraventricular conduction delay (IVCD), have been described [15,23,24,25].

Subanalyses of landmark CRT trials unanimously show that patients with baseline LBBB benefit more from CRT. However, there are various definitions for LBBB [15,25,26]. Moreover, van Stipdonk et al. [27] showed that there is considerable intra- and interobserver variability in applying the various LBBB criteria. In a study examining 316 CRT patients, baseline ECGs of patients were analyzed using six distinct LBBB classifications, as defined by AHA/ACC/HRS (American Heart Association/American College of Cardiology/Heart Rhythm Society), ESC (European Society of Cardiology) 2006, ESC 2009, ESC 2013, and the classification proposed by Strauss and colleagues [28]. In the multivariate analysis, a significant association with the combined endpoint was observed only for ESC 2009 and 2013 classifications and for Strauss [28]. Looking beyond LBBB, although the response to CRT is less in RBBB and IVCD patients, selected patients may also have a delayed LV lateral wall (LVLW) activation that can be corrected with CRT and could, thus, benefit equally with respect to clinical response [29]. Different activation patterns can be observed in LBBB depending on the anatomy of the conduction system or the location of the block, which cannot be evaluated with surface ECG, but also LV hypertrophy or fibrosis can cause wide QRS morphology that meets LBBB criteria [30]. Also, patients with RBBB masking the LBBB pattern have been shown to have activation delay similar to that in LBBB [31]. As a consequence, patient selection using conventional ECG parameters may lead to missing a substantial number of patients with delayed LV activation who may actually benefit from CRT.

**Figure 1:**
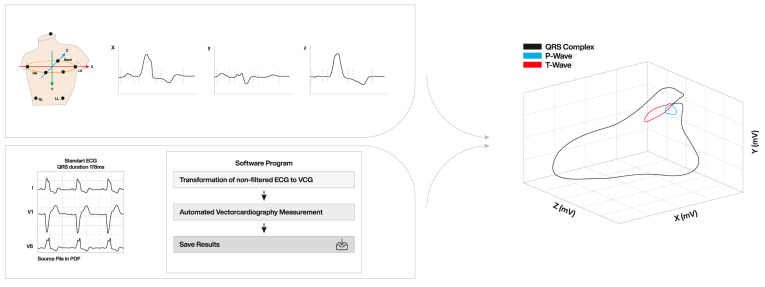
3D vector loop created from standard 12-lead ECG and vector conversion to 3 orthogonal leads (X-, Y-, and Z). ECG: electrocardiography; LA: left arm; LL: left leg; RA: right arm; RL: right leg VCG: vectorcardiography.

The potential of the 3D VCG to be a superior tool, as compared to the ‘flat’ 12-lead ECG, especially in the field of CRT, has been recognized in recent years. The most commonly used conventional vectorcardiographic method is the 8-electrode system according to Frank [32]. With the increasing interest in vectorcardiography and due to the limited availability of Frank-VCG recording systems, software programs have now been developed to create a VCG from the standard 12-lead ECG. Through a mathematical conversion, the 3D loop can be created from a digital 12-lead ECG (Figure 1).

Engels et al. [33] compared the Frank-VCG and the Kors-VCG in CRT patients specifically. They showed that both methods have comparable results. Variables calculated from Frank- and Kors-VCG were found to show correlation coefficients between 0.77 and 0.90 [33]. In a study examining pre-implantation ECG recordings of 864 CRT patients, fully automatically calculated QRS area and manually calculated QRS area values were compared [34]. The automated and manual analyses yielded highly comparable results (hazard ratios (HR) 2.09 vs. 2.03, respectively) [34]. Automatic measurement of QRS area not only creates ease of use of this parameter in clinical practice but also facilitates the analysis of large-scale studies.

The VCG provides additional information that cannot be revealed in the standard 12-lead ECG. It provides information about the amplitude and duration of both depolarization and repolarization. Unlike ECG parameters, evaluation of VCG parameters is not dependent on observer interpretation, as it contains quantitative information on both depolarization and repolarization, regardless of the direction or manner of ventricular activation. QRS area and T-wave area, QRS- and T-wave axes in three dimensions, spatial QRS- and T-wave integrals, and spatial angle between the QRS- and T-wave axes can be calculated with VCG [17,19,35,36].

**Figure 2:**
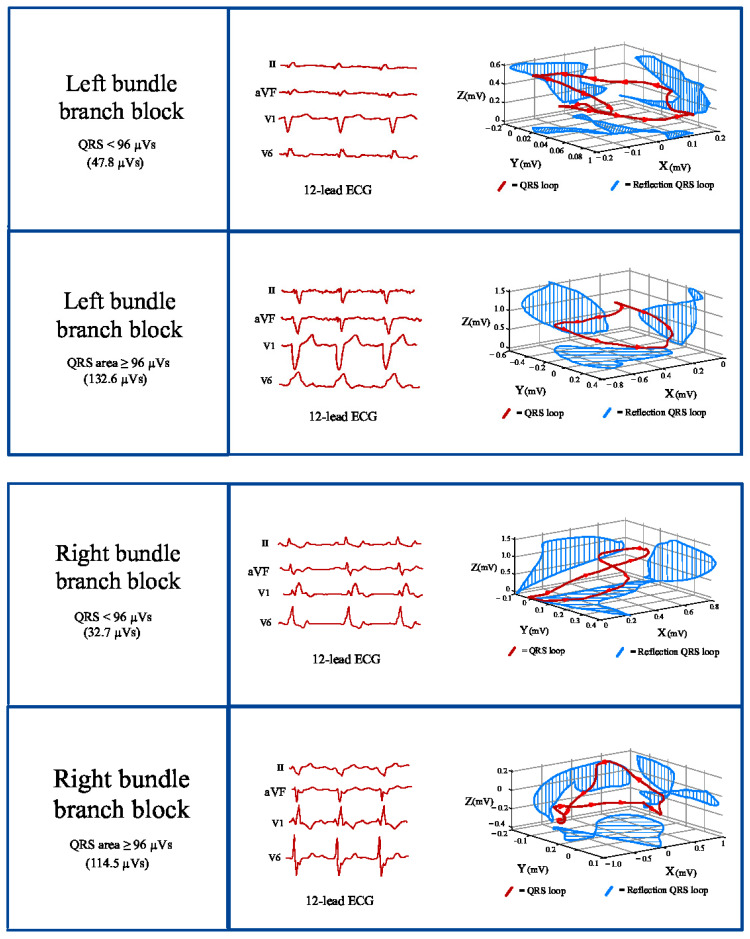
Conversion of the 12-lead ECG to a 3D orthogonal lead vector in X-, Y-, and Z-axes. ECG: electrocardiography.

Currently, the most commonly reported VCG parameter for identifying patients who could benefit from CRT is the QRS area (Figure 2). The QRS area, which provides 3D information about the electrical forces within the heart, is calculated as the sum of the area under the QRS complex in the calculated X-, Y-, and Z-lead [QRS area = (QRS_area,x_^2^ + QRS_area,y_^2^ + QRS_area,z_^2^)^1/2^] [17,18]. The 3D area of the QRS loop (QRS area) is assumed to reflect the unopposed electrical forces during ventricular depolarization. It provides quantitative information about ventricular activation, taking into account the direction and form of ventricular activation, both in the presence of native conduction and pacing. Furthermore, unlike QRS duration and morphology, the calculation of QRS area is not dependent on the observer’s interpretation.

In earlier studies, the conventional VCG was shown to have a higher sensitivity and specificity than the standard 12-lead ECG in the diagnosis of left ventricular hypertrophy [37]. In addition, it appeared to be superior to the ECG in the diagnosis of chamber enlargement and associated with the presence of electrically inactive areas in patients with myocardial infarction [38,39,40]. With respect to conduction delay, the value of VCG-derived markers has only recently been evaluated.

## 3. Vectorcardiographic QRS Area and CRT

As a VCG parameter, the QRS area holds substantial significance within the context of CRT. It uniquely integrates two crucial aspects of ventricular depolarization: amplitude and duration. Electrical dyssynchrony in HF patients, which can be corrected through CRT, would result in substantial unopposed electrical forces during ventricular depolarization. It has been suggested that these forces could be effectively quantified by the QRS area, which represents the area of the QRS complex across the three principal axes. The utilization of the QRS area provides the advantage of objective quantification, thereby eliminating reliance on subjective evaluations of notching or slurring in certain ECG leads. Furthermore, it mitigates the potential inaccuracies associated with improper ECG lead placement, ensuring greater consistency and reliability in measurement.

In patients with HF and ventricular conduction disturbance, the appropriate substrate for CRT is considered to be delayed activation of the LV lateral wall (LVLW) resulting in LV mechanical dyssynchrony, and BiV (fusion) pacing from the LV lateral wall and RV apex has been shown to improve this dyssynchrony [41,42]. Mafi-Rad et al. [43] examined whether the QRS area could identify delayed LVLW activation by performing coronary venous electroanatomical mapping in 51 CRT candidates. They demonstrated that the QRS area is superior in identifying delayed LVLW activation than QRS duration and LBBB morphology [43]. Van Deursen et al. [44] found that the QRS area was larger in LBBB patients than in IVCD patients. In the same study, the lower QRS area in patients with ischemic cardiomyopathy compared to non-ischemic ones can be explained by the fact that electrically non-conductive scar tissue is more common in ischemic hearts. Nguyên et al. [45] investigated the association between VCG parameters and myocardial scar on cardiac magnetic resonance (CMR) imaging in 32 CRT patients. It was demonstrated that the QRS area is inversely associated with focal scar, indicating that myocardial scar leads to a smaller QRS area. Although CRT provides improvements in dyssynchrony by applying electrical therapy, the underlying myocardial properties also play an important role in the response to treatment. It is conceivable that the above-mentioned aspects of VCG-derived markers could aid in improving patient selection in CRT by incorporating both electrical and myocardial features in a single marker.

The first study examining the association between QRS area and CRT outcomes was conducted by Van Deursen et al. [44] (Table 1). In this prospective cohort of 81 CRT patients, QRS area, QRS duration, and QRS morphology were studied on echocardiographic response to CRT. The QRS area was shown to be a good parameter in predicting echocardiographic response to CRT with an odds ratio (OR) of 10.2 and a 95% confidence interval (CI) of 3.4 to 31.1. This OR was higher than that for the QRS duration (OR = 2.5) and QRS morphology (OR = 5.5) [44]. Tereshchenko et al. [46] investigated the association between the vectorcardiographic sum of the absolute QRST integral (SAI) and echocardiographic response to CRT in 234 patients. Patients with high SAI (3rd tertile) had 2.5-times greater odds of response than those with low SAI (1st tertile) and 1.9-times greater than the lower two tertiles combined. They demonstrated that SAI is an independent predictor (beyond QRS duration and BBB morphology) of the echocardiographic response to CRT (OR, 1.87 [1.03–3.42]; *p* = 0.041) [47]. In the prospective Markers And Response to CRT (MARC) study [47], the predictive value of a set of clinical, electrical, structural, and blood biomarkers for echocardiographic reverse remodeling was evaluated in patients with a CRT indication. From this multimarker evaluation, the CAVIAR (CRT-Age-Vectorcardiographic QRS area-intraventricular mechanical delay-apical rocking) score was derived and found to be predictive of LVESVi reduction (effect estimate −10.4, 95% CI (−14.9%, −5.8%), *p* < 0.0001) [47]. From these markers, the QRS area contributed the most to the predictive value of the model, whereas QRS duration and morphology were not (independently) associated [47]. Van Stipdonk et al. [17], in their study examining 1.492 CRT patients, evaluated the value of the QRS area compared with QRS duration and morphology in the association with clinical and echocardiographic outcomes. The combined clinical outcome of all-cause mortality, cardiac transplantation, and left ventricular assist device (LVAD) implantation was significantly lower in patients with a QRS area ≥ 109 μVs as compared to patients with a QRS area < 109 μVs (HR, 0.49 [0.41–0.59]). In both patients with class I indications and those without class I indications, echocardiographic response rates were significantly higher in patients with QRS area ≥ 109 μV than in those with QRS area < 109 μV (OR, 3.54 [2.38–5.28]; *p* < 0.001 and OR, 1.90 [1.19–3.03]; *p* = 0.009, respectively). It was found that the association of QRS area with all outcomes was stronger than that of LBBB morphology and QRS duration separately and at least as strong as their combination [17]. The QRS area improved the identification of patients amenable to CRT, compared to QRS morphology and QRS duration, with respect to survival free from cardiac transplantation and LVAD implantation (AUC, 0.61 versus 0.55 and 0.51, respectively; *p* < 0.001) and echocardiographic reduction in LVESV (AUC, 0.69 versus 0.58 and 0.58, respectively; *p* < 0.001). The QRS area was the only independent electrocardiographic determinant associated with the clinical endpoint (HR, 0.50 (0.35–0.71)). Moreover, in non-LBBB patients (ESC class IIa/IIb indication for CRT), the QRS area was able to differentiate patients surviving, free from heart transplantation and LVAD implantation, whereas QRS duration was not [17]. Similarly, the QRS area was significantly associated with echocardiographic response, but the QRS duration was not in these non-LBBB patients [17]. Strengthening these results, in a study by Emerek et al. [48] examining 705 CRT patients, the QRS area was found to be associated with clinical outcomes, not only in those with QRS duration ≥ 150 ms (unadjusted HR: 3.85, *p* < 0.001) but also in those with QRS duration < 150 ms (unadjusted HR: 1.76, *p* < 0.001). The group of non-LBBB patients (class IIa/b indications) is a particular group of interest, as current CRT implantation practice is very heterogeneous. In a study of 790 non-LBBB patients, the association between many ECG parameters, such as QRS morphology, QRS duration, PR interval, left ventricular activation time (LVAT), and fragmented QRS (fQRS), as well as the QRS area with both clinical and echocardiographic response to CRT, was evaluated [18]. Only the QRS area (HR 2.33 [1.44, 3.77], *p* = 0.001) and PR interval (HR 2.03 [1.51, 2.74], *p* < 0.001) showed an independent association with the clinical outcome in the multivariable regression analysis [18]. The findings of these studies together demonstrate that the QRS area has a consistent significant association with clinical outcomes across all CRT indication classes.

Whereas the aforementioned studies confirm the potential role of the QRS area in measuring dyssynchrony and, hence, patient selection for CRT, its continuous nature may also allow it to play a role in the quality of resynchronization in patients treated with CRT. Okafor et al. [49] investigated the change in QRS area after CRT (ΔQRS area) and its association with cardiac and all-cause mortality, heart failure hospitalizations, and arrhythmic events in 380 CRT patients. ΔQRS area ≥ 45 µVs after CRT predicted a lower rate of cardiac mortality (HR: 0.19), total mortality (HR: 0.50), total mortality and heart failure hospitalizations (HR: 0.44), and the occurrence of arrhythmic events (HR: 0.26; *p* < 0.001). A study by Ghossein et al. [50] examining 1.299 CRT patients confirmed this association with both clinical and echocardiographic outcomes. Multivariate regression analysis revealed that ΔQRS area ≥ 62 μVs remained the strongest predictor of clinical and echocardiographic outcome [50]. Thus, in addition to using the baseline QRS area in patient selection, targeting a greater reduction in the QRS area via CRT optimization at implantation or thereafter may provide additional benefit to patients. 

In addition to patient selection, procedural delivery characteristics play an important role in increasing the benefit derived from CRT. Proper LV lead placement (LVLP) is a key element of successful CRT implantation. It is recommended to place the LV lead fluoroscopically in the non-apical posterolateral region [15]. However, studies using advanced cardiac imaging modalities such as echocardiography, electrocardiographic imaging (ECGi), and CMR suggest that detailed electrical and myocardial properties at different pacing positions on the LV lateral wall may influence outcomes significantly [51,52,53,54,55]. However, these modalities do not provide real-time information about the extent of resynchronization achieved at the time of implantation at a particular LV lateral wall pacing site. Interventricular conduction delay during the procedure has been shown to be a predictor of CRT response and can be useful in the selection of CRT modality [56]. In a study, CRT outcomes were evaluated by comparing the interventricular conduction delays-guided resynchronization group (DRG) with the standard CRT group (SRG) [57]. In the DRG group, if the time interval between the sensed RV and sensed LV (RVs-LVs) measured in sinus rhythm was ≥100 ms or the paced RV to sensed LV interval (RVp-LVs) was ≥120 ms, the coronary sinus lead was left in its original position; otherwise, conduction system pacing (CSP) was applied [57]. They demonstrated that the DRG-based algorithm significantly reduced the incidence of the primary outcome, a composite of cardiovascular deaths, HF hospitalizations, and urgent unplanned clinic visits at the 1-year follow-up [57]. However, the presence of patients with different baseline conduction abnormalities (LBBB, RBBB, and atrioventricular block linked to reduced EF) reduced the specificity of the findings for the separate patient groups targeted for resynchronization.

As the QRS area provides potentially real-time (12-lead ECG derived) feedback on the quality of resynchronization during the implantation procedure, it allows for optimization of the LV lead position. In a study conducted by Okafor et al. [58], the association between the ΔQRS area and acute hemodynamic response (AHR) was evaluated in CRT patients. In 26 patients, the maximum rate of change of LV pressure (ΔLV dP/dtmax) during CRT implantation with LV pacing at different LV lead positions was assessed [58]. The ΔQRS area was found to be a significant predictor of ΔLV dP/dtmax after CRT (AUC 0.81; *p* < 0.001) [58]. Also, it was found that LV leads placed in a scarred LV segment were associated with a lower ΔQRS area (+22.2 ± 58.4 μVs, *p* < 0.001) in comparison to LV leads placed away from the scar or with no scarring at all (−3.28 ± 38.1 μVs and −43.8 ± 36.8 μVs, *p* < 0.001). Ghossein et al. [59] evaluated a total of 188 different pacing sites in 52 CRT patients for the association with AHR as measured by ΔLV dP/dtmax and ΔQRS area. They found that the ΔQRS area was significantly associated with AHR at different LV pacing sites, with a strong correlation between AHR and QRS area (median R = 0.76, IQR 0.35; 0.89) [59]. These results show that, indeed, the QRS area may potentially be used to guide LVLP in CRT.

## 4. Vectorcardiographic T-Wave Area and CRT Response

The QRS area as well as QRS morphology and QRS duration reflect the depolarization phase. However, repolarization is also significantly changed in the presence of ventricular dyssynchrony and may provide further information regarding the electrical substrate amenable to CRT and the extent of resynchronization derived from CRT.

Like depolarization, repolarization is sensitive to the structural changes that occur in heart failure, like an increase in collagen content, loss of myofibrils, and disruption of gap junctions [60,61]. Moreover, the pathophysiological changes in HF cause prolongation of the action potential, which not only causes prolongation of repolarization but also results in the dispersion of repolarization [60]. Changes that may occur in ventricular repolarization can be evaluated by analyzing T-wave and QT interval properties on a 12-lead ECG [62]. An association of T-wave morphology descriptors with prognosis in patients with systolic HF was shown before [63]. Total cosine between QRS and T-wave was found to be a significant risk stratifier (OR: 0.40, *p* = 0.003) of cardiovascular mortality in HF patients with reduced LVEF [63]. T-wave alternans (TWAs), characterized by subtle beat-to-beat variations in T-wave observed on ECG during gradual heart rate increases induced by exercise or atrial pacing, has been shown to be associated with the dispersion of repolarization and sudden cardiac arrest [64]. Data on repolarization parameters in patients undergoing CRT implantation are scarce. Anh et al. [65] examined the effects of different pacing modalities on the T-wave in patients receiving CRT. Biventricular pacing reduced both T-wave alternans (TWAs) and T-wave amplitude more than RV pacing and LV pacing [65]. Another study observed that among HF patients with LBBB, those characterized by larger T-wave morphology dispersion, larger T-wave loop area, and more negative total cosine between QRS and T-wave values, as assessed on digital standard 12-lead ECG, demonstrated superior echocardiographic responses to CRT [66]. There are, however, no clear criteria for T-wave morphology and duration, like there are for QRS morphology and duration.

When evaluated with VCG, the direction, amplitude, and duration of the repolarization vector as well as the T-wave area can be calculated quantitatively [67,68]. There are much less data on T-wave area and CRT response than there are for QRS area (Table 2). A study by Engels et al. [69] examining 244 CRT patients showed that the T-wave area was associated with echocardiographic response in patients with baseline LBBB. Patients with T-wave area above the median value were found to have a higher increase in LVEF (11.3 ± 9.1% vs. 6.1 ± 9.7%, *p* < 0.01) following CRT [69]. The T-wave area was found to be a predictor of echocardiographic response (OR per 10 μVs increase 1.172 [*p* < 0.001]) [69]. The same group subsequently examined the association between the baseline T-wave area and clinical outcomes in another cohort of 335 CRT patients [70]. The baseline T-wave area was smaller in the patient group that experienced the primary composite outcome of HF hospitalization, HTx, LVAD implantation, or death (74 ± 45 μVs) than in those without the primary composite outcome (88 ± 47 μVs). They evaluated the patients by grouping them according to the median T-wave area and QRS morphology. During a mean follow-up of 2.4 years, the primary composite outcome was less common (*p* < 0.01) in the patient subgroup with a large T-wave area and LBBB (36%) than in patients with LBBB and a small T-wave area or non-LBBB patients with a small or large T-wave area (48, 57, and 51%, respectively) [70]. In a multivariable analysis, a large T-wave area and LBBB were the only independent predictors of the primary composite outcome besides high creatinine levels and use of diuretics [70]. More recently, a large, multicenter study of 1.355 CRT recipients confirmed the association of baseline T-wave area to both clinical and echocardiographic outcomes [19]. During a mean follow-up of 3.7 years, the clinical composite outcome of a combination of all-cause mortality, heart transplantation, and left ventricular assist device implantation was significantly less commonly seen [27% vs. 37%, HR 0.66 (0.54–0.81), *p* < 0.001)], and the echocardiographic response was significantly larger (26 ± 30% vs. 9 ± 31%, *p*< 0.001) in those with a baseline T-wave area ≥ 66 μVs [19]. The baseline T-wave area was found to be an independent predictor of both clinical and echocardiographic outcomes [HR 0.46 (0.31–0.69), *p* < 0.001 and OR 3.10 (2.02–4.76), *p* < 0.001, respectively] [19]. Moreover, the association between the QRS area and the T-wave area was further deepened. A significant correlation was found between T-wave area and QRS area (*p* < 0.001, r = 0.783) [19]. Patients were divided into four groups based on T-wave area (≥66 μVs vs. <66 μVs) and QRS area (≥109 μVs vs. <109 μVs), and LVESV reduction was found to be larger in patients with QRS area ≥109 μVs and T-wave area ≥ 66 μVs, as compared with the other groups (*p* < 0.001) [19]. The event-free survival rate was higher in patients with both QRS area ≥ 109 μVs and T-wave area ≥ 66 μVs [*n* = 616, HR 0.47 (0.38–0.58), *p* < 0.001] and patients with QRS area ≥ 109 μVs and T-wave area < 66μVs [*n* = 100, HR 0.35 (0.21–0.56), *p* < 0.001], compared with the other two subgroups [19]. When the QRS area was added to the prediction model, the association between T-wave area and echocardiographic response remained [OR 2.0 (1.20–3.36), *p* = 0.008]; however, the association with clinical outcomes did not [HR 0.68 (0.40–1.14), *p* = 0.143] [19]. These results indicate that the baseline T-wave area may have additional value in the identification of responders to CRT when added to the QRS area.

## 5. Vectorcardiography in Conduction System Pacing

Interest in conduction system pacing (CSP), particularly left bundle branch area pacing (LBBAP), in the setting of CRT has exponentially increased in recent years. It may further increase the benefit from CRT in selected patients by providing more physiological pacing of the LV [71]. However, a major concern for the use of CSP in CRT is its restricted ability to restore proximal conduction disturbances rather than distal or diffuse conduction disturbances, which are also often co-existing in CRT-eligible patients. As this ability to amend the baseline conduction disturbance is the most important determinant of success for this new pacing mode, vectorcardiographic markers of the extent and level of conduction disease and the extent of resynchronization achieved may play an important role in evaluating patients eligible for and treated with CRT by means of CSP.

**Figure 3:**
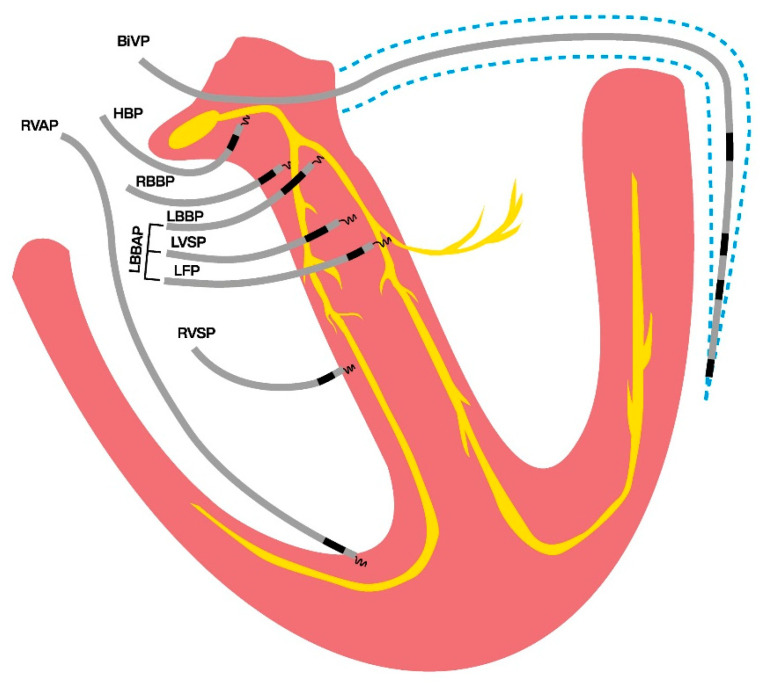
Conduction system pacing locations in and nearby the heart’s conduction system, in contrast to traditional pacing modalities; right ventricular- and biventricular pacing. BiVP: biventricular pacing; HBP: his bundle pacing; LBBAP: left bundle branch area pacing; LBBP: left bundle branch pacing; LFP: left fascicular pacing; LVSP: left ventricular septal pacing; RBBP: right bundle branch pacing; RVAP: right ventricular apical pacing; RVSP: right ventricular septal pacing.

As CSP encompasses several pacing locations in the actual conduction system of the heart or nearby (Figure 3), the evaluation of the properties of each site to enable true synchronous pacing has been evaluated recently. In a single-center study examining 80 patients who underwent LBBAP for a brady-pacing indication, electrocardiographic characteristics were evaluated during intrinsic rhythm, right ventricular septum pacing (RVSP), and LBBAP [72]. The increase in QRS area from baseline intrinsic (narrow QRS; QRS area 32 ± 16 μVs) activation was found to be significantly higher in RVSP (73 ± 20 μVs) than in LBBAP (41 ± 15 μVs) [72]. Also, the QRS area was significantly smaller in patients with actual LBB capture compared to patients without LBB capture (43 ± 18 μVs vs. 54 ± 21 μVs) [72]. Liu et al. [73] evaluated the electrocardiographic and vectorcardiographic characteristics of pacing from different branches of the left bundle conduction system in 91 patients who had a brady-pacing indication. They found that the paced QRS area in pacing the left bundle trunk (LBTP) was similar to that during intrinsic rhythm (35.1 ± 15.8 μVs vs. 34.7 ± 16.6 μVs, *p* = 0.98), whereas in the left bundle fascicular pacing (LBFP), the paced QRS area was significantly larger compared to intrinsic rhythm (43.4 ± 15.8 μVs vs. 35.7 ± 18.0 μVs, *p* = 0.01) [73]. Heckman et al. [74] investigated VCG parameters during pacing at different depths within the septum in 50 patients undergoing LBBAP for a bradycardia indication and structurally normal hearts. They found that the QRS area significantly decreased from 82 ± 29 μVs during RVSP to 46 ± 12 μVs with LVSP and 38 ± 15 μVs with LBBP [74]. In addition, the QRS area during LBBAP (41 ± 14 μVs) was not significantly different from that during intrinsic activation (35 ± 19 μVs) [74]. In contrast, QRS duration was found to increase significantly during LBBAP (118 ± 24 ms) compared to intrinsic activation (96 ± 11 ms). This is probably due to local myocardial capture and delayed activation of the RV, which manifests as an RBBB pattern on the ECG during LBBAP.

These findings suggest that the QRS area may be an appropriate indicator for the assessment of the extent of synchronous pacing in CSP. It is conceivable that VCG can be used in evaluating the success of the procedure, in lead localization, and even in post-procedural optimization in LBBAP-CRT.

## 6. Clinical Implications and Future Perspectives

Studies conducted in recent years have revealed very promising results regarding the value of VCG-derived parameters, especially in patient selection for CRT. VCG-derived parameters appear to be superior to standard 12-lead ECG parameters, QRS duration and morphology, with respect to reproducibility, association with other measures of dyssynchrony, and their association with outcomes in patients treated with CRT. Moreover, there is a sound physiological theoretical explanation for a superior association with the success of CRT treatment. Standard ECG equipment and computer software are sufficient to convert the standard 12-lead ECG to VCG. And, as such, VCG parameters can easily be made available for use in clinical practice.

Evidence also suggests that, in addition to patient identification for CRT, VCG-derived parameters could serve as a marker for the optimization of CRT therapy with respect to LV (and CSP) lead placement but also in post-procedural programming of CRT pacing. However, future prospective studies, especially in CSP-facilitated CRT, are needed on this subject. Evidence needs to be strengthened with prospective studies examining short- and long-term outcomes of the use of VCG derived parameters in this area.

## 7. Conclusions

In recent years, there has been a large interest in VCG-derived parameters for the purpose of the optimization of CRT. Increasing evidence suggests that VCG parameters, especially the QRS area and T-wave area, are useful in the identification of patients with a substrate amenable to CRT. Studies show that the baseline QRS area (and, to a lesser extent, T-wave area) has a strong association with both clinical and echocardiographic outcomes to CRT. Moreover, evidence suggests that the extent of change in these markers is independently associated with the response to CRT, with a possible role in guiding implantation and optimization of CRT. More data are needed to establish the role of VCG-derived markers in all of the mentioned fields for implanting clinicians to embrace these easily implementable markers in global CRT practice.

## Figures and Tables

**Table 1:** Studies investigating QRS area, QRS duration, and morphology in relation to CRT response.

Study	Design	Patients (*n*)	Inclusion	Follow-Up	Outcomes	Parameters	Results
Van Deursen, et al. [44] (2014)	Prospective	81	Class I and II	6 mo	Echocardiographic response (LVESV reduction ≥ 15%)	- QRS area > 98 μVs - QRS duration > 156 ms - LBBB • Conventional • AHA • Strauss et al.	- OR 10.2; 95% CI: 3.4–31.1 - OR 2.5; 95% CI: 0.9–6.6 - LBBB • OR 5.5; 95% CI: 0.9–32.4 • OR 4.5; 95% CI: 1.6–12.6 • OR 10.0; 95% CI: 3.2–31.1
Mafi-Rad, et al. [43] (2016)	Prospective	51	Class I and II	-	Delayed LV activation (>75% of QRS duration)	- QRS area - QRS area > 69 μVs - QRS duration - LBBB (ESC)	- AUC 0.89; 95% CI: 0.79–0.99 - Sens: 87%; spec: 92% - AUC 0.49; 95% CI: 0.33–0.65 (ns) - Sens 76%; spec 100%
Nguyen, et al. [45] (2018)	Prospective	33	Class I and II	6 mo	Echocardiographic response (LVESV reduction ≥ 15%)	- QRS area - QRS duration	- AUC 0.74; 95% CI: 0.56–0.91 - AUC 0.64; 95% CI: 0.44–0.83 (ns)
MARC Study [47] (2018)	Prospective	213	Class I and II	6 mo	- Echocardiographic response (LVESVi reduction)	- QRS area - QRS duration - LBBB	* Adjusted effect estimate - 10.4%, 95% CI: (−14.9, −5.8), *p* < 0.001 - ns - ns
Van Stipdonk, et al. [17] (2018)	Retrospective	1.491	Class I and II	3.4 yrs	A. Echocardiographic response (LVESV reduction ≥ 15%) B. Clinical outcome-All-cause mortality C. Clinical outcome-HF hospitalization	1. QRS area quartiles 2. LBBB + QRSd	A-1. ** OR 1.65; 95%CI: 1.43–1.90 2. OR 1.29; 95%CI: 1.09–1.52 B-1. ** HR: 0.75; 95% CI: 0.69–0.83 2. HR: 0.93; 95% CI: 0.69–1.02 (ns) C-1. HR: 0.76; 95% CI: 0.60–0.96 2. HR: 0.96; 95% CI: 0.71–1.21 (ns)
Emerek, et al. [48] (2019)	Retrospective	705	Class I and II	3.1 yrs	All-cause mortality	QRS area ≤ 95 μVs	*** HR: 1.65; 95% CI: 1.25–2.18
Okafor, et al. [49] (2019)	Retrospective	380	Class I and II	3.8 yrs	Cardiac mortality	1. QRS area 2. QRS duration 3. LBBB	1. † HR: 0.99; 95% CI: 0.98–0.99 2. † HR: 1.01; 95% CI: 0.99–1.02 (ns) 3. HR: 0.79; 95% CI: 0.79–1.27 (ns)
Dural, et al. [18] (2021)	Retrospective	790	Class II (non-LBBB)	3.7 yrs	A. Clinical outcome-All-cause mortality B. Echocardiographic response (LVESV reduction ≥ 15%)	1. QRS area ≥ 109 μVs 2. QRSd ≥ 150 ms 3. RBBB 4. IVCD	A-1. ** HR: 2.33; 95% CI: 1.44–3.77 2. ns 3. ns 4. ns B-1. OR: 2.00; 95% CI: 1.09–3.66 2. ns 3. ns 4. ns
Ghossein, et al. [50] (2021)	Retrospective	1.299	Class I and II	3.9 yrs	A. Clinical outcome-All-cause mortality B. Echocardiographic response (LVESV reduction ≥ 15%)	1. QRS area ≥ 109 μVs 2. QRSd ≥ 150 ms 3. LBBB (ESC)	A-1. ‡ HR: 0.72; 95% CI: 0.56–0.96 2. ‡ HR: 1.1; 95% CI: 0.89–1.49 (ns) 3. ‡ HR: 0.78; 95% CI: 0.61–1.00 (ns) B-1. ‡ OR: 1.7; 95% CI: 1.2–2.5 2. ‡ OR: 1.4; 95% CI: 0.97–2.0 3. ‡ OR: 1.8; 95% CI: 1.2–2.7

* Adjusted for sex, age, iCMP, NYHA class, LBBB, kidney function, various ECG variables, and various mechanical dyssynchrony variables, including variables from the CAVIAR score (see text). ** Multivariable model for QRS area, QRS duration, and morphology. *** Adjusted for QRS duration and morphology, age, sex, ischemic heart disease, first degree atrioventricular block, atrial fibrillation/flutter, LV ejection fraction, NYHA functional class, kidney function, cerebrovascular disease, chronic lung disease, and medical therapy for heart failure. † Multivariable model with QRS area, QRS duration, morphology, and other ECG variables, and baseline characteristics including age, sex, and iCMP. ‡ Adjusted for QRS duration/morphology, age, sex, atrial fibrillation, iCMP, kidney function, and diabetes mellitus. AHA: American Heart Association; AUC: area under the curve; CI: confidence interval; CRT: cardiac resynchronization therapy; ECG: electrocardiogram; ESC: according to European Society of Cardiology; HF: heart failure; HR: hazard ratio; iCMP: ischemic cardiomyopathy; IQR: interquartile range; IVCD: non-specific intraventricular conduction delay; LBBB: left bundle branch block; LV: left ventricle; LVESV: left ventricular end-systolic volume; LVESVi: left ventricular end-systolic volume index; ns: non-significant; NYHA: New York Heart Association classification of HF; OR: odds ratio; RBBB: right bundle branch block.

**Table 2:** Studies investigating T-wave area, QRS duration, and morphology in relation to CRT response.

Study	Design	Patients (*n*)	Inclusion	Follow-Up (Years)	Outcomes	Parameters	Results
Engels, et al. [69] (2015)	Retrospective	244	Class I and II	6 mo	Echocardiographic response (LVEF increase ≥ 5%)	- T-wave area, μVs - QRS duration, ms - QRS area, µVs	- * OR: 1.17; 95% CI: 1.08–1.25 - OR: 0.99; 95% CI: 0.92–1.08 (ns) - OR: 1.12; 95% CI: 1.05–1.19
Vegh, et al. [70] (2016)	Retrospective	335	Class I and II	2.4 yrs	All-cause mortality	- T-wave area, μVs - QRS duration > 150 ms - QRS area, μVs - LBBB	- ** HR 0.63; 95% CI: 0.42–0.96 - ns - ns - HR 0.65; 95% CI: 0.44–0.98
Dural, et al. [19] (2024)	Retrospective	1.355	Class I and II	3.7 yrs	A. All-cause mortality B. Echocardiographic response (LVESV reduction ≥ 15%)	1. T-wave area, μVs 2. QRS duration, ms ≥ 150 ms 3. LBBB (ESC)	A-1. *** HR 0.46; 95% CI: 0.31–0.69 2. HR 0.72; 95% CI: 0.47–1.10 (ns) 3. HR 1.15; 95% CI: 0.75–1.77 (ns) B-1. *** OR: 3.10; 95% CI: 2.02–4.76 2. OR: 1.34; 95% CI: 0.83–2.18 (ns) 3. OR: 2.34; 95% CI: 1.37–4.00)

* Multivariable model for T-wave area, QRS area, and QRS duration. ** Multivariable model for T-wave area, QRS area, QRS duration, and QRS morphology. *** Multivariable model for T-wave area, QRS duration, and QRS morphology. CI: confidence interval; ESC: according to European Society of Cardiology; HR: hazard ratio; iCMP: ischemic cardiomyopathy; LBBB: left bundle branch block; LVEF: left ventricular ejection fraction; LVESV: left ventricular end-systolic volume; ns: non-significant; OR: odds ratio.

## Data Availability

Not applicable.
